# Fluoxetine improves bone microarchitecture and mechanical properties in rodents undergoing chronic mild stress – an animal model of depression

**DOI:** 10.1038/s41398-022-02083-w

**Published:** 2022-08-20

**Authors:** Raymond W. Lam, Hee-Kit Wong, Ramruttun A. Kumarsing, Anna N. Chua, Roger C. Ho, Roger S. McIntyre, Cyrus S. Ho

**Affiliations:** 1 Department of Orthopaedic Surgery, Yong Loo Lin School of Medicine, National University of Singapore, 119228 Singapore, Singapore; 2 Department of Psychological Medicine, Yong Loo Lin School of Medicine, National University of Singapore, 119228 Singapore, Singapore; 3 Institute for Health Innovation and Technology (iHealthtech), National University of Singapore, 117599 Singapore, Singapore; 4 Mood Disorders Psychopharmacology Unit, University of Toronto, Toronto, ON Canada; 5 University Health Network, 399 Bathurst Street, MP 9-325, Toronto, ON M5T 2S8 Canada; 6 Department of Pharmacology and Toxicology, University of Toronto, Toronto, ON Canada; 7 Department of Psychiatry, University of Toronto, Toronto, ON Canada; 8 Brain and Cognition Discovery Foundation, Toronto, ON Canada

**Keywords:** Physiology, Depression

## Abstract

Depression is one of the most prevalent mental disorders associated with reductions in bone mineral density and increased fracture risk. Fluoxetine is a highly prescribed selective serotonin reuptake inhibitor (SSRI) in the treatment of depression and is reported to be a risk factor for fractures. The present study examined the effect of fluoxetine on bone microarchitecture and the mechanical properties under chronic mild stress (CMS), a rodent model of depression. Thirty-one 6–9 week-old rats were allocated to 4 groups: 1) CMS + fluoxetine group (*n* = 10), 2) fluoxetine-only group (*n* = 5), 3) CMS + placebo group (*n* = 10) and 4) control group (no CMS and treatment) (*n* = 6). After 16 weeks, bone microarchitecture of the distal femur was analyzed by µCT. Mechanical properties were assessed by the three-point bending test, and antidepressant efficacy was determined by sucrose preference and forced swimming tests. Significant correlations were found between volume of sucrose intake and bone volume/tissue volume (BV/TV) (*p* = 0.019) and elastic absorption energy (*p* = 0.001) in the fluoxetine only group. The fluoxetine-only group showed significantly higher in the second moment of area in y-direction (*p* = 0.0298), horizontal outer diameter (mm) (*p* = 0.0488) and average midshaft thickness (mm) (*p* = 0.00047) than control group. Comparing with the control group, there was a significant reduction in trabecular number (Tb.N) in the CMS + fluoxetine group (*p* = 0.026) but not the fluoxetine-only group (*p* > 0.05). Significant increases in trabecular separation were observed in the metaphysis of CMS + placebo (*p* = 0.003) and CMS + fluoxetine (*p* = 0.004) groups when compared to the control group but not in the fluoxetine-only group (*p* > 0.05). During the three-point bending test, the fluoxetine-only group demonstrated significantly higher structural strength than controls (*p* = 0.04). Micro computed tomography (µCT) slices showed loss of trabecular bone in the metaphysis region of the CMS + fluoxetine and CMS + placebo groups but not the fluoxetine-only and control groups. In an animal model of depression, the adverse effect on the bone microarchitecture was caused by CMS but not by fluoxetine. Without exposure to CMS, fluoxetine significantly increased the cross-sectional area, trabecular bone area, structural strength and osteoblasts / bone area as compared to control condition.

## Introduction

Major depressive disorder (MDD) is one of the most prevalent psychiatric conditions, which affects approximately 16% of the U.S. adult population. Schweiger and colleagues published the first study examining the relationship between depression and bone mineral density (BMD) in 1994; it showed a 15% drop in bone mineral density in 70 subjects. One retrospective study and four prospective studies found significant positive associations between depression and risk of fractures [1].

Selective serotonin reuptake inhibitors (SSRI) inhibit serotonin reuptake and increase serotonin (5-hydroxytryptamine, 5-HT) levels, a neurotransmitter implicated in the pathogenesis of major depressive disorder and suicide [2]. Fluoxetine is an antidepressant of the SSRI class. Fluoxetine is the first-line pharmacological management of depression and has high popularity index [3]. Aside from activity on the nervous system, fluoxetine was found to inhibit osteoblast [4] and osteoclast [5] differentiation. Due to the popularity of SSRI usage, there have been a growing number of research studies which have demonstrated a correlation between its usage and non-vertebral fracture risk [6]. Tsapakis et al. (2012) reported that in vitro, in vivo and clinical research data indicate that SSRIs have a negative effect on bone at the therapeutic dose [7]. The proportion of fracture among patients who consumed low dose SSRI was 28% [8] and the clinical fracture risk for older adults who consumed SSRI was 2 fold [9]. The higher doses of SSRI may further increase fracture risk in dose-dependent manner [10, 11]. Verdel et al. (2010) proposed the antidepressant’s affinity for the serotonin receptors might contribute to the increased risk of osteoporotic fractures [12]. Wu et al. [13] conducted a meta-analysis and concluded that SSRIs may increase risk of fracture, independent of depression and BMD. Tsapakis [7] advised doctors and patients to be cautious about adverse skeletal effects of SSRIs. The aforementioned recommendation needs to be balanced against the benefits of SSRI therapy in relieving depressive symptoms and improvement of functioning [14], as it may cause more worry among people with depression and lower their adherence to SSRI therapy. In contrast, other studies did not find the association between antidepressant use and fracture. Spangler et al. (2008) and Hlis et al. (2018) reported minimal association between depressive symptoms and changes in either BMD [15] or fracture risk [16]. Furthermore, Spangler et al. (2008) and Ho et al. (2022) also found that SSRI use was not associated with changes in BMD [16, 17],

Some of the population-based and prospective cohort studies were not primarily designed to evaluate the effect of SSRIs on BMD and fracture risk [18]. Patient ages were above 50 in 10 out of 13 studies that demonstrated SSRI increased fracture risk [19] and this finding could be confounded by osteoporosis. For premenopausal women with depression, Aydin et al. [18] and Ho et al. [17] reported that SSRI treatment decreased bone resorption and increased bone formation. Such excessive bone formation may lead to overly dense bone mineral density and increase the risk of fracture as observed in the osteopetrosis. Human studies are prone to confounding factors, such as smoking, substance abuse, variations within the depression period, adherence and dosage of SSRIs, body mass index, ethnicity, diet, presence of other medications, which may affect BMD [20]. In addition, the possibility of bone fracture due to other concomitant medication and/or comorbidity needs to be adjusted for [21]. Van den Brand et al. (2009) proposed that further studies are required to elucidate the mechanistic changes in bone after SSRI use [22]. Therefore, a suitable animal model is needed to provide the means to test the correlation in a controlled environment.

The effect of fluoxetine on bone has been studied in genetic knock-out [23], adolescent [24], menopausal and inflammatory models [25]. 5-HTT knock-out mice displayed a consistent skeletal phenotype of reduced mass, altered architecture, and inferior mechanical properties. Lipopolysaccharide (LPS) was used to establish a depression model in rats [26] Under inflammation, LPS, a key constituent of gram-negative bacteria, induce osteoclast formation and promote bone resorption. LPS injection into mice resulted in net bone loss, whereas a net gain in bone mass was seen when LPS was given together with fluoxetine. The foregoing study suggested that inflammation plays important roles in bone loss but it can be reversed by fluoxetine (SSRI). This may also apply to people with depression.

The chronic mild stress (CMS) paradigm has become a widely adopted animal model for major depressive disorder (MDD) for four decades [27]. Previous studies validated the CMS paradigm to study its effects on bone architecture and mechanical properties [28–30]. We hypothesize that fluoxetine would not cause bone loss and biomechanical deterioration in rats without exposure to the CMS, which is analogous to the human depressive state [31]. The aims of this study were to 1) evaluate the bone microarchitecture change by µCT and histological method; and 2) perform a biomechanical study on the femur and tibia bone after 16 weeks of CMS, CMS + fluoxetine, CMS + placebo and control condition.

## Materials and methods

### Study design and animals

Thirty-one Sprague-Dawley rats were used in this study. The sample size was based on our research budget to maintain the number of rats in the animal research facility throughout the study period and previous studies using the CMS to elicit biological changes in rats [32]. The inclusion criteria included female gender and the age was 6–8-week-old. As depression is more prevalent in women [33] and previous research focused on the fracture risk of women who suffered from MDD and consumed SSRI [34], this study included female rats only. The thirty-one rats were randomly divided into four groups: 1) the control group, which did not involve any treatment or lavage, 2) the fluoxetine-only group, which was treated with fluoxetine (10 mg/kg/day) by oral gavage for 16 weeks without any CMS treatment [27], 3) the CMS + placebo group, which was treated with CMS and oral gavage with 0.5 ml of distilled water on a daily basis, and 4) the CMS + fluoxetine group, which was chronically stressed and treated by oral gavage with fluoxetine (10 mg/kg/day) for 16 weeks (Table 1). Our fluoxetine dosage (10 mg/kg) is 2 times higher than a previous study (5 mg/kg) [35]. Each rat was housed separately in a ventilated cage with free access to pelleted rodent diet and water ad libitum and 12 h light/day cycle. All experiments were conducted according to institutional guidelines and were approved by the Institutional Animal Care and Use Committee (IACUC) at the National University of Singapore. Every effort was made to minimize the number and suffering of animals.

**Table 1:** The grouping of rats (*n* = 31).

Group	Condition/Treatment	No. of Rats
Control	No treatment and lavage	6
Fluoxetine Only	Lavage with fluoxetine (10 mg/kg)	5
CMS + Placebo	Chronic mild stress + gastric lavage of distilled water	10
CMS + Fluoxetine	CMS and lavage with fluoxetine (10 mg/kg)	10

### The Chronic Mild Stress (CMS) procedure

The rats were adapted to their new surroundings for three days before they were subjected to any procedure or treatment. Twenty rats were subjected to the CMS procedure [36] for 16 weeks; modifications were based on previous research [31] and recommendations from the local ethics committee. Each rat from the CMS group was subjected to one stressor per day in addition to continuous single housing. The stressors were: [1] food deprivation for 18 h; [2] continuous overnight illumination; [3] soiled cage with 100 ml of water spilled onto the bedding for 6 h; [4] cold water swimming at 18 ˚C for 5 min; [5] shaking on a rocking bed with an orbital motion of 200 rpm for 15 min; [6] physical restraint for 20 min; and [7] water deprivation for 18 h. The body weights of all rats were measured on a weekly basis.

### The sucrose preference test

The sucrose preference test was performed by blinded research assistants before any treatment and after 16 weeks of the CMS procedure to operationally define anhedonia [31, 36, 37]. Each rat was subjected to three 1 h training sessions before any treatments. The training consisted of making a selection from either 1% (w/v) sucrose solution or distilled water presented to them, succeeding 23 h of food and water deprivation [36, 38]. At the end of each test, both distilled water and sucrose intake were calculated by measuring the differences of respective pre- and post-weighed bottles. The sucrose preferences (SP) were calculated in accordance to the following ratio: SP = sucrose intake/ (sucrose intake + water intake). The final training test (Day 8) was considered as the baseline of sucrose preference test. The sucrose preference test was conducted again in the 16 weeks for all rats.

### The Forced Swim Test (FST)

The Forced Swim Test (FST) is a standard test to evaluate learned helplessness in rats and was performed by blinded research assistants. A 15 min training session under similar conditions was included and conducted 24 h before the test. Rats were dropped individually into a vertical plexiglass cylinder filled with 23–25 ˚C of water to a depth of 15 cm. The duration of immobility was recorded after the rats had acquired an immobile posture upon initial vigorous activity (2 min).

### Morphological and topological characteristics of bone (µCT)

After 16 weeks of treatment, rats were sacrificed. Both femurs were harvested for biomechanical study, µCT and histology. Femur bone microarchitecture was studied using micro-computed tomography (µCT) scanning. The proximal femur, including the femoral head and the metaphysis of the dissected femurs, were scanned using micro-XCT Xradia Inc, Canada (100 KV x-ray source with a focal spot size of 5 µm). The scans consisted of 1024 slices with 10 µm (nominal resolution) spanning a 10.24 mm of each bone. The specimen was stably fixed on the scanning fixture before it was placed on the specimen stage of the micro-XCT for scanning. Scanning of the specimen was carried out at 60 keV, 8 W, and 133 µAmp. Two areas within the femur were selected: 1) Distal metaphysis section and 2) Mid-epiphysis section. For the distal metaphysis section, 100 slides were included. The region of interest was selected by polygon tools and interpolated. After interpolation, cortical bones were removed by altering the ROI shape. Bone volume/tissue volume (BV/TV, %), bone surface/bone volume (BS/BV %), bone surface/tissue volume (BS/TV %), trabecular number (Tb.N) and trabecular separation (Tb.Sp, μm) were measured. The trabecular thickness (Tb.Th) was measured by CTAn, version 1.13 (64 bit) Skyscan software, Belgium.

### Bone mechanical testing

Three-point bending and femoral fracture tests were used to determine several important biomechanics parameters; the elastic stiffness, fracture load, and elastic absorption energy were determined from the curve of three-point bending and femoral fracture compression test. The setup followed the design by Clarisa Bozzini et al. [39]. Femurs were thawed at room temperature before mechanical testing. Each bone was secured on the two lower supports of the anvil of an Instron universal testing machine. Preload was 1 N, loading rates were 5 mm/min, and length span was 14 mm. The femoral fracture test was performed according to the methods. The femoral bone lengths were measured and potted with dental cement. The following parameters were use: Loading rate 2.0 mm/min, compression force = Force x sin α bending force = Force x cosα bending moment = bending force x d.

### Histology and osteoblast density

Femurs were fixed by formalin and dehydrated by gradient ethanol, embedded in Technovit 7200 resin, according to the hard tissue histology protocol. Longitudinal sections were cut with a high-speed water-cooled precision saw (EXAKT 300CP Band System, Norderstedt, Germany) into parallel sections of 30 μm thickness. The slides were stained with basic fuchsin and counterstained with methylene blue metachromatic dye in order to differentiate the connective tissues (e.g., bone, bone marrow, cartilage and fibrous tissue). Histological evaluation was performed in order to compare bone microarchitecture differences among the control and other groups. An overall view was taken by AZ100, Nikon, Japan at magnification factor of 3X. Osteoblast density was measured by using an inverted optical microscope (Olympus IX71, Osaka, Japan).

### Cytokine analysis

The peripheral blood serum concentrations of IL-1β, IL-6, and IL-17 were quantified by following instructions from the commercially available multiplex bead-based immunoassay (EMD Millipore Catalogue RECYTMAG-65K). All samples were assayed in duplicates. Briefly, the serum samples were thawed to 4 ˚C and centrifuged for 3 mins at 1000 x *g* to settle any residue. Following this, an immunoassay was performed with beads coated with anti- IL-1β, IL-6, and IL-17 antibodies that were added after the addition of 25 μl of standards and samples into specific wells of microtiter filter plates. This was followed by 2 h incubation at room temperature with shaking at 450 rpm on the plate shaker. The plate was then washed and incubated for 1 h at room temperature with detection antibodies, followed by the addition of streptavidin-phycoerythrin for another 30 mins of incubation. The plate was then washed twice and sheath fluids were added to all wells. Subsequently, the plate was read with the Luminex 200 plate reader; standard curves of IL-1β, IL-6, and IL-17, ranging from 2.4 to 73.2 pg/ml up to 10,000–300,000 pg/ml, were automatically constructed via the five-parameter logistic method. The cytokine concentrations of experimental samples were calculated based on the standard curve generated.

### Statistical analysis

Data from μCT, osteoblast density and biomechanical testing were analyzed by using Origin Pro 9.0. All normally distributed data were reported as the means with estimate of variation as standard deviation (SD). If the data were not normally distributed, the Mann Whitney test was used to compare median values among groups. *P* < 0.05 was considered as significant. Pearson correlation was performed between behavioral data (i.e., volume of sucrose intake and immobility time during the forced swim test) and bone histomorphometry and biomechanics parameters. Two-way ANOVA with post-hoc tests were used to compare bone parameters and variance among the four groups. We also studied the interaction between CMS and fluoxetine on different bone parameters.

## Results

### General observations on body weight

Table 2 compares the change of body weight from baseline to week 16 in the 4 groups of rats. Using the control group as a reference, the fluoxetine-only group (−34.0%) and the CMS + placebo (−27.6%) group showed less weight gain but not statistically significant (*p* > 0.05). The CMS + fluoxetine group demonstrated significantly lower body weight when compared to the controls (−40.7%, p = 0.011).

**Table 2:** Comparison of body weight of the control group versus the fluoxetine-only, CMS + placebo and CMS + fluoxetine groups at 4 months.

	Control (*n* = 6)	Fluoxetine-only (*n* = 5)	CMS + placebo (*n* = 10)	CMS + fluoxetine (*n* = 10)
Mean weight change (grams)	67.4 ± 19.6	44.5 ± 14.2	48.8 ± 28.6	40.0 ± 13.5
*p*-value		0.083	0.221	0.011^*^

^*^P < 0.05.

### Depression behavior measurement

**Fig. 1:**
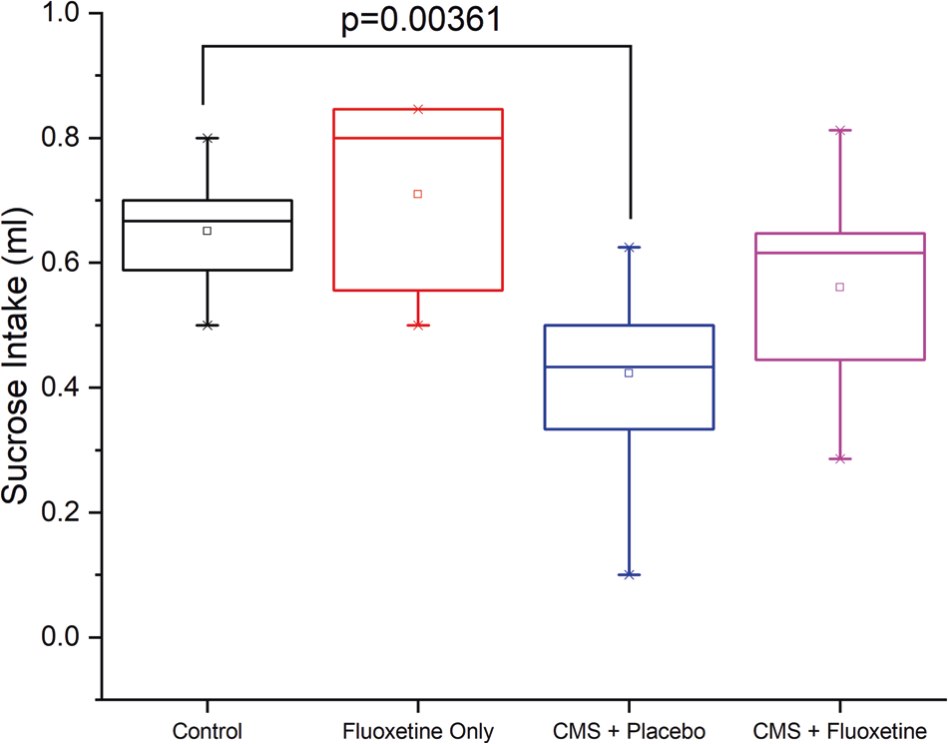
Evaluation of the antidepressant effects of an antidepressant by the sucrose intake test in rats. Rats received sucrose solution volume (ml) control, fluoxetine only, CMS + placebo and CMS + fluoxetine groups.

Figure 1 compares the sucrose intake of rats in week 16 among 4 groups of rats. The CMS + placebo group demonstrated the lowest volume of sucrose water intake among 4 groups and it was significantly lower than controls (*p* = 0.0036). This suggests that the CMS + placebo group demonstrated the lowest level of appetite that is a depressive symptom.

**Fig. 2:**
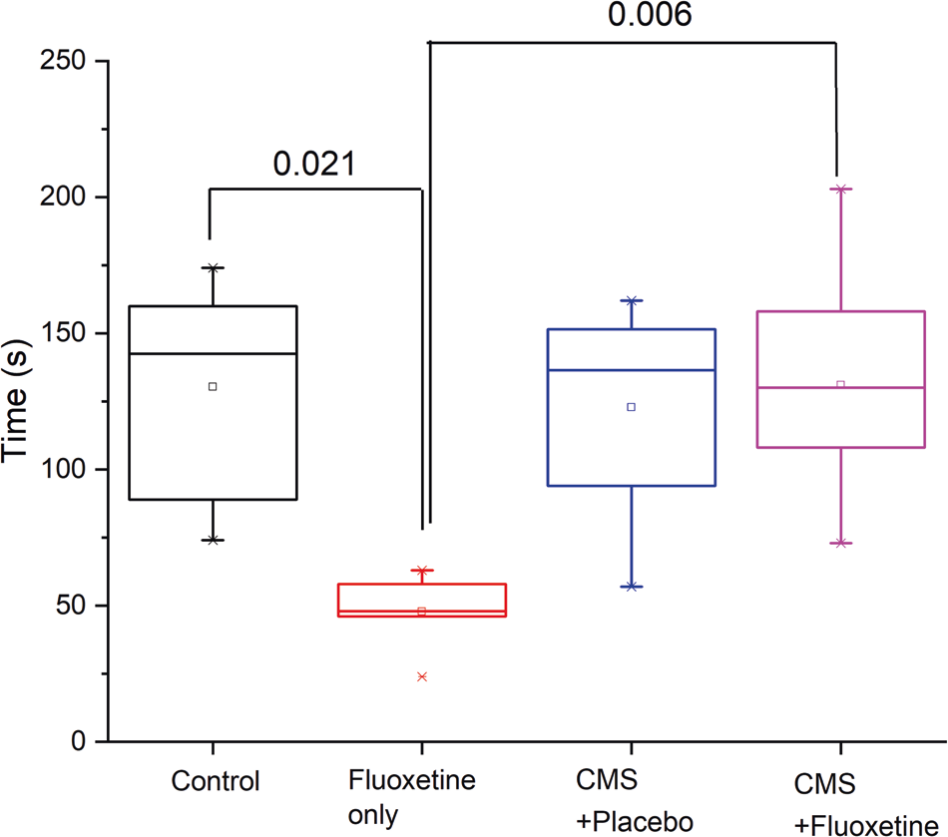
Immobile time under the forced swimming test (FST) in the control, fluoxetine only, CMS + placebo and CMS + fluoxetine groups.

Figure 2 compares the mean immobility time among the 4 groups of rats during the forced swim test (FST). The CMS + placebo group demonstrated the longest mean immobility time, while the CMS + fluoxetine group demonstrated the shortest mean immobility time. The immobility time was significantly lower in the fluoxetine group as compared to the control group (*p* = 0.021), suggesting the activation effect of fluoxetine.

Regarding the correlation between behavioral data and bone histomorphometry parameters, there were no significant correlations between volume of sucrose intake and bone histomorphometry parameters (*p* > 0.05) except significant correlation was found between volume of sucrose intake and bone volume/tissue volume (BV/TV) (correlation coefficient −0.938, *p* = 0.019) in the fluoxetine only group, suggesting higher oral intake was associated with higher BV/TV in the fluoxetine-only group (See Supplementary Table 1). There were no significant correlations between immobility time during FST and bone histomorphometry parameters (*p* > 0.05) except significant correlations were found between immobility time and trabecular separation (Tb.Sp) (correlation coefficient = −0.709, *p* = 0.049) in the CMS + placebo group as well as between immobility time and trabecular number (Tb.N) (correlation coefficient = 0.798, *p* = 0.032) in the CMS + fluoxetine group (See Supplementary Table 2). There were no significant correlations between sucrose intake volume and bone biomechanics parameters (*p* > 0.05) except significant correlation was found between volume of sucrose intake and elastic absorption energy (correlation coefficient = 0.99, *p* = 0.001) in the fluoxetine-only group. suggesting higher oral intake was associated with higher elastic absorption energy in the fluoxetine-only group (See Supplementary Table 3). There were no significant correlations between immobility time during FST and bone biomechanics parameters (*p* > 0.05) (See Supplementary Table 4).

### Cytokine analysis

For cytokine analysis, there were no significant differences in IL-1β, IL-6, and IL-17 levels (4-week vs. baseline; 16-week vs baseline) in CMS-fluoxetine, CMS-placebo and control groups (*p* > 0.05) (See Supplementary Table 5).

### Morphological and topological characteristics of bone

**Fig. 3:**
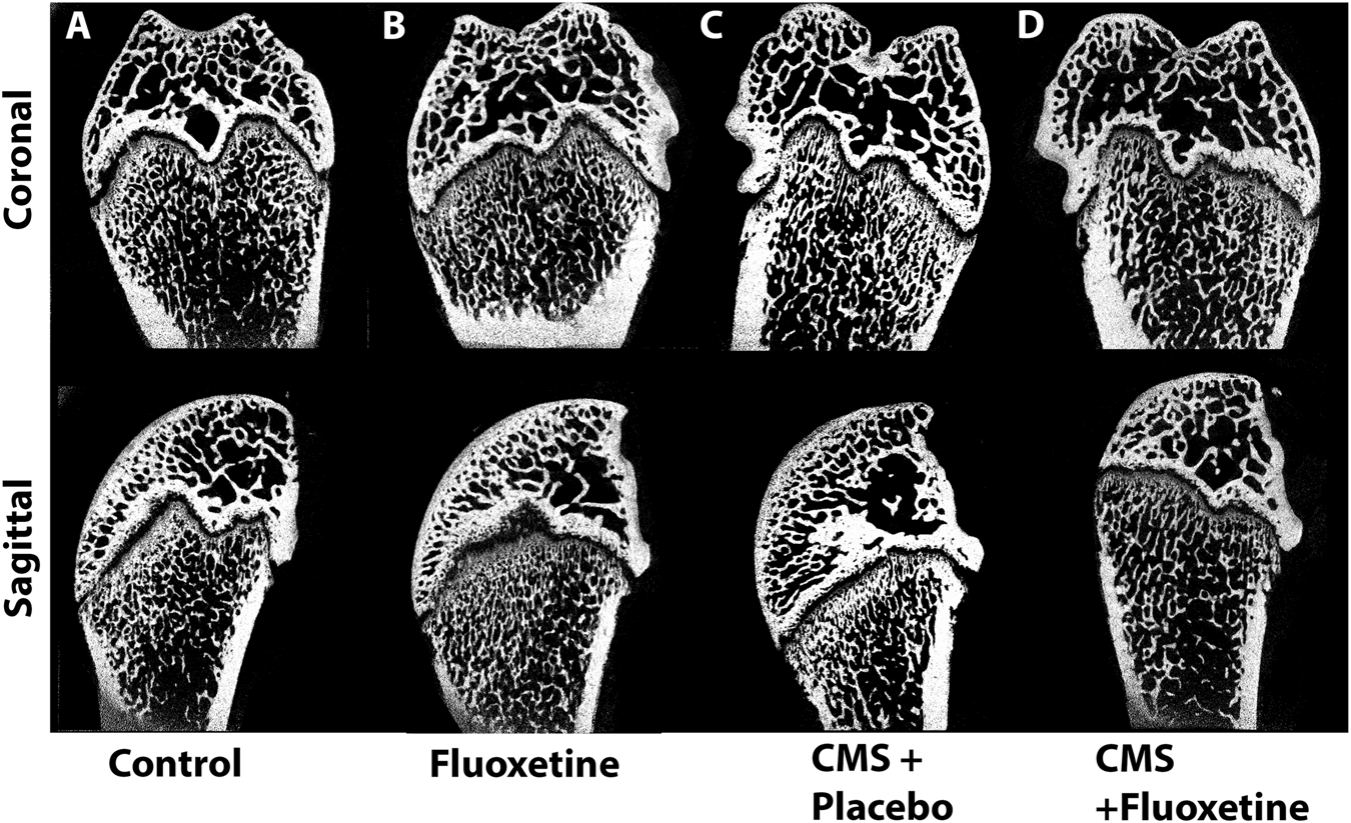
Representative micro computed tomography (μCT) 2D longitudinal sections of rat femurs. **A** Control, **B** fluoxetineonly, **C** CMS + placebo, and **D** CMS + fluoxetine groups.

Figure 3 illustrates reconstructed images from x-ray µCT slices depicting trabecular structure of the 4 groups. The CMS + placebo group shows loss of trabecular bone structure. Further loss of trabecular bone is observed in the metaphysis region of the CMS + fluoxetine group rat femur (see Fig. 3) as compared to the control and fluoxetine-only groups. The images are matched with the result of the CTAn data.

**Fig. 4:**
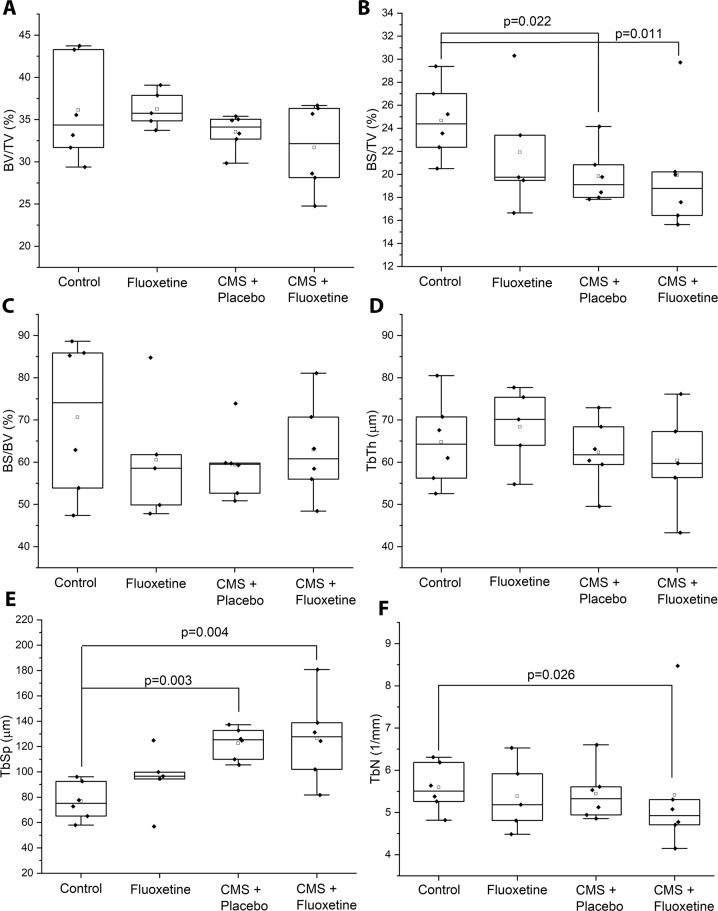
Histomorphometry parameters of metaphysis trabecular bone. **A** Bone Volume/Tissue Volume (BV/TV). **B** Bone Surface/Tissue Volume (BS/TV), **C** Bone surface/Bone Volume BS/BV. **D** Trabecular thickness. **E** Trabecular Separation. **F** Trabecular number among 4 treatment groups: 1) control, 2) fluoxetine only, 3) CMS + placebo and 4) CMS + fluoxetine groups.

Figure 4 compares the femur microarchitecture parameters after 16 weeks of experiment. Using the control group as the reference, there were no significant differences in bone volume/tissue volume (BV/TV) in the fluoxetine-only group, CMS + placebo group and CMS + fluoxetine group (*p* > 0.0.5). There was no interaction effect between CMS and fluoxetine on BV/TV (F = 1.37, *p* = 0.25) for all groups. Using the control group as the reference, CMS + placebo group (−15.0%, *p* = 0.022) and CMS + fluoxetine group (−10.2%, *p* = 0.011) showed significantly lower bone surface/tissue volume (BS/TV). There was no significant difference in BS/TV between fluoxetine-only and control groups (*p* > 0.05). There was no interaction effect between CMS and fluoxetine on BS/TV (F = 0.64, *p* = 0.43) for all groups. There was no significant difference in bone surface/bone volume (BS/BV) among 4 groups and no interaction effect between CMS and fluoxetine on BS/BV for all groups. (F = 2.63, *p* = 0.12). Using the control group as the reference, there were no significant differences with the fluoxetine-only, CMS + placebo and CMS + fluoxetine groups in the trabecular thickness (Tb.Th) (*p* > 0.05). There was no interaction effect between CMS and fluoxetine on Tb.Th for all groups (F = 0.88, *p* = 0.36). There were significant increases in trabecular separation (Tb.Sp) in the CMS + placebo (+54.2%, *p* = 0.003) and CMS + fluoxetine (+63.7%, *p* = 0.004) groups when compared to the control group. There was no significant difference in Tb.Sp between fluoxetine-only and control groups (*p* > 0.05). There was no interaction effect between CMS and fluoxetine on trabecular separation Tb.Sp (F = 0.72, *p* = 0.40). In the comparison of the trabecular number (Tb.N) using the control group as the reference. there was a significant reduction in the Tb.N in the CMS + fluoxetine (−14.2%, *p* = 0.026) but there was no significant difference with the fluoxetine only group (*p* > 0.05). There was no interaction effect between CMS and fluoxetine on TbN for all groups (F = 0.008, *p* = 0.92).

### Bone mechanical testing

The fluoxetine-only group showed significantly higher in the second moment of area in y-direction (*p* = 0.0298), horizontal outer diameter (mm) (*p* = 0.0488), and average midshaft thickness (mm) (*p* = 0.00047) than control group (See Table 3). There were no significant differences in cross section area, second moment of area in x-direction, polar moment of inertia in z-direction, vertical outer diameter, horizontal outer diameter, vertical inner diameter, horizontal inner diameter and average midshaft thickness between the control group and other three groups (*p* > 0.05) (See Table 3).

**Table 3:** Comparison of geometric parameters of the femur among four groups.

Group	Cross section area (mm^2^)	Second moment of area in x-direction Ix (xCSMI) (mm^4^)†	Second moment of area in y-direction Iy (yCSMI) (mm^4^) †	Polar moment of inertia in z-direction Iz = Ix + Iy (mm^4^) †	Vertical outer diameter (mm)	Horizontal outer diameter (mm)	Vertical inner diameter (mm)	Horizontal inner diameter (mm)	Average midshaft thickness (mm)
Control group (No CMS + No oral gavage)	6.32 ± 0.49	4.69 ± 0.83	7.13 ± 0.83	11.82 ± 1.50	3.01 ± 0.22	3.78 ± 0.13	1.46 ± 0.39	2.23 ± 0.23	0.78 ± 0.10
Fluoxetine-only group (10 mg/kg)	9.37 ± 0.31 (*p* = 0.0797)	6.22 ± 0.29 (*p* = 0.4407)	12.04 ± 1.00* (*p* = 0.0298)	18.08 ± 1.25 (*p* = 0.0599)	3.05 ± 0.09 (*p* = 0.8711)	4.36 ± 0.12* (*p* = 0.0488)	0.81 ± 0.23 (*p* = 0.0504)	2.12 ± 0.28 (*p* = 0.9465)	1.12 ± 0.13** (*p* = 0.00047)
CMS + placebo group	7.55 ± 1.19 (*p* = 0.7534)	5.28 ± 1.51 (*p* = 0.9179)	9.10 ± 3.20 (*p* = 0.5128)	14.38 ± 4.63 (*p* = 0.6248)	3.01 ± 0.23 (*p* = 0.9999)	3.97 ± 0.44 (p = 0.7534)	1.11 ± 0.38 (*p* = 0.3009)	2.07 ± 0.45 (p = 0.9057)	0.95 ± 0.13 (*p* = 0.0506)
CMS + Fluoxetine group (10 mg/kg)	7.19 ± 1.45 (*p* = 0.7216)	5.20 ± 2.00 (*p* = 0.9096)	8.97 ± 3.37 (*p* = 0.5682)	14.18 ± 5.19 (*p* = 0.6838)	2.96 ± 0.42 (p = 0.9904)	3.99 ± 0.48 (*p* = 0.7216)	1.18 ± 0.41 (*p* = 0.4922)	2.21 ± 0.56 (*p* = 0.9997)	0.89 ± 0.13 (*p* = 0.2976)

*p < 0.05, **p < 0.001, ^†^Explanation of Ix, Iy and Iz can be found in Supplementary Fig. 1.

From Table 4a the fluoxetine-only group demonstrated significantly higher in structural strength as compared to controls during the three-point bending test (*p* = 0.04). There were no significant differences between controls and other groups in elastic stiffness and elastic yield force (*p* > 0.05). For bone mechanical testing data, there was no interaction effect between CMS and fluoxetine on elastic stiffness (F = 0.01, *p* = 0.92), structural strength (F = 2.64, *p* = 0.12), elastic absorption energy (F = 0.15, *p* = 0.7), elastic yield force (F = 0.27, *p* = 0.6) and elastic yield deflection (F = 1.48, *p* = 0.23).

**Table 4:** Comparison of the biomechanical properties of the femur (three-point bending test) among four groups.

Group	Elastic Stiffness (N/mm)	*p*-value	Structural Strength	*p*-value	Elastic Absorption Energy	*p*-value	Elastic Yield Force		Elastic Yield deflection	
Control (No CMS + No oral gavage)	589.58 ± 37.55		210.83 ± 24.91		18.13 ± 9.79		124.77 ± 45.88		0.32 ± 0.13	
Fluoxetine-only (10 mg/kg)	604.83 ± 26.12	0.7152	231.80 ± 11.20	0.0449*	18.65 ± 2.86	0.3810	147.52 ± 11.04	0.1464	0.28 ± 0.04	0.5828
CMS + placebo	567.70 ± 81.23	0.9999	204.66 ± 46.95	0.8519	16.85 ± 5.90	0.8122	134.96 ± 29.65	0.6147	0.28 ± 0.06	0.972
CMS + fluoxetine group	601.39 ± 36.59	0.6733	211.43 ± 31.93	0.9775	18.16 ± 4.89	0.9638	151.08 ± 15.33	0.4866	0.33 ± 0.09	0.6504

*p < 0.05.

### Histology and osteoblast density

**Fig. 5:**
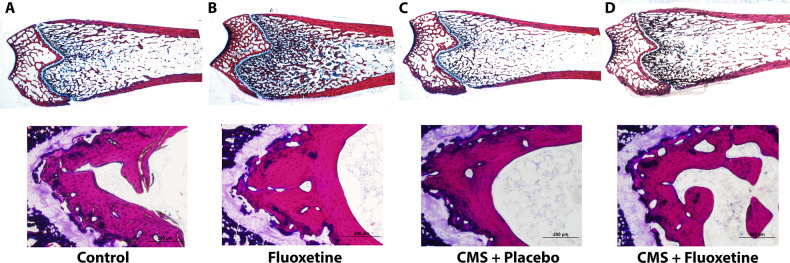
Representative histological sections (sagittal view) of bone femur specimens stained with basic fuchsin and counterstained with methylene blue. **A** Control, **B** Fluoxetine-only, **C** CMS + placebo and **D** CMS + fluoxetine (Mag 40X) groups.

**Fig. 6:**
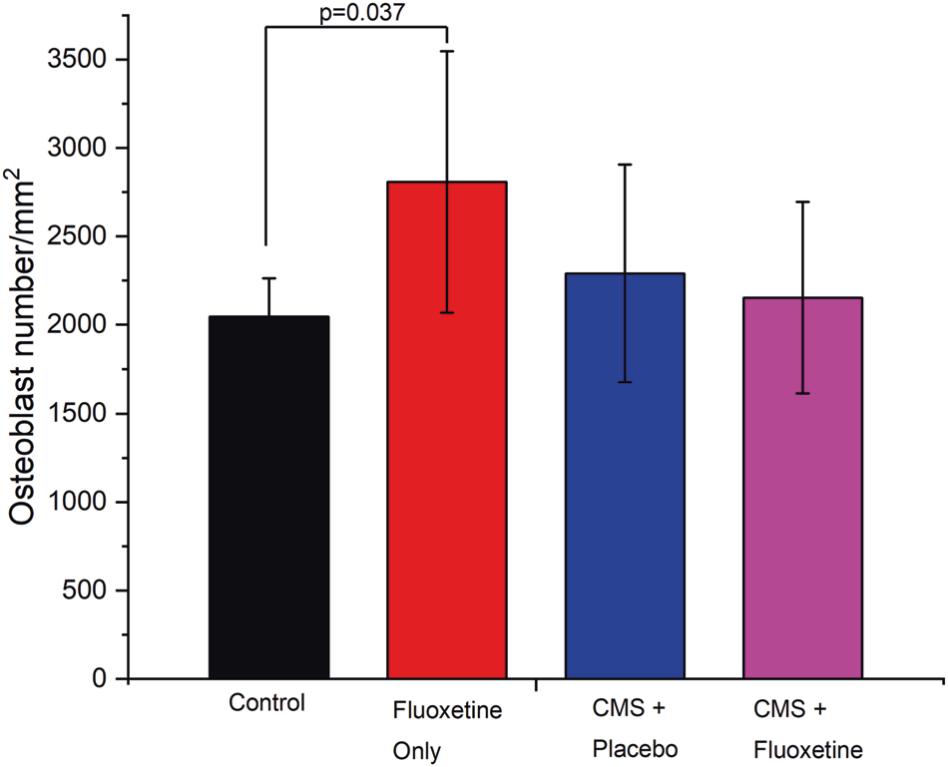
Osteoblast number within metaphysis region. (1) Control (No CMS + No oral gavage), (2) fluoxetine-only, (3) CMS + placebo and (4) CMS + fluoxetine groups.

Figure 5 compares the trabecular bone volume in the mid-diaphyseal region among the 4 groups of rats. The fluoxetine-only group demonstrated significantly higher trabecular bone area in comparison to the three other groups. There was no significant difference in the trabecular bone volume among the control, CMS + placebo and CMS + fluoxetine groups. The average midshaft thickness of the femur was in the order of fluoxetine > CMS + placebo > CMS + fluoxetine > control (See Table 3), which matched the micro-CT data. Figure 6 shows that the fluoxetine-only group demonstrated significantly higher osteoblasts/per bone area (mm^2^) in comparison to the controls ( +7.5%, *p* = 0.037).

## Discussion

Depression due to chronic stress is an important inducer of bone loss and microarchitecture deterioration [40–42]. To validate the CMS rodent model, sucrose water intake and forced swimming test was performed. The CMS + placebo group demonstrated the lowest volume of sucrose water intake among the 4 groups. CMS + placebo group exhibited the lowest level of appetite, which is a symptom of depression. The CMS + fluoxetine group showed some restoration of appetite. The CMS + fluoxetine group demonstrated significantly lower body weight when compared to the controls. This finding is not surprising because a meta-analysis reported that fluoxetine is associated with weight loss in clinical patients [43]. In the fluoxetine-only group, the significant correlation between sucrose intake and BV/TV and elastic absorption energy suggests that higher oral intake is associated with stronger bone and better bone function in rats exposed to fluoxetine. During the FST, the CMS + placebo group demonstrated the longest mean immobility time, which is a symptom of depression. In contrast, the CMS + fluoxetine group demonstrated lower immobility time than CMS + placebo group. This demonstrates that the fluoxetine treatment relieve depression in rodent model.

Under stress free conditions, fluoxetine had a positive effect on the bone microarchitecture. The fluoxetine-only group demonstrated significantly higher second moment of area in y-direction, horizontal outer diameter, average midshaft thickness, trabecular bone area, structural strength and osteoblasts / bone area as compared to control group. µCT slices showed loss of trabecular bone in the metaphysis region of the CMS + fluoxetine and CMS + placebo groups but not the fluoxetine-only and control groups. The fluoxetine-only group demonstrated significantly higher structural strength than controls. There were no other significant differences in femur microarchitecture parameters between the fluoxetine-only and control groups suggesting that detrimental effects were mainly associated with CMS. Both CMS + placebo and CMS + fluoxetine showed significant increase in trabecular separation in the metaphysis region compared to the control. The results are consistent with the clinical report by Kinjo et al. [44] who did not find the use of antidepressants to have any association to reduction in femur BMD in 14,646 healthy adults.

Our results are in accordance with Diem et al. (2013) who reported that SSRI was not associated with an increase in bone loss in hips and femoral necks of middle-aged women [45]. Our results showed that fluoxetine-only group demonstrated significantly higher osteoblast/per bone area in mm^2^ than controls. This finding further affirmed previous in-vitro studies which found that citalopram, an SSRI antidepressant enhanced osteoblast proliferation and did not affect mineralization in a cell-line system [46].

### Limitations

This study has several limitations. Due to the limited budget, there were fewer rats in the control and fluoxetine-only groups as compared to the CMS-placebo and CMS-fluoxetine groups. This had resulted in unbalanced distribution in terms of the number of rats per group. The resources to measure plasma cortisol, which is one of the factors that causes bone loss, levels were not available. Yazici [47] reported that there was no correlation between plasma cortisol levels, duration of depression, antidepressant use and BMD in premenopausal women. Moreover, the activity level of rats was not measured. Nevertheless, Warden et al. [20] demonstrated that the skeletal effects of psychotropic drugs did not result from animal physical inactivity.

### Clinical significance

This study has two important clinical implications about the association between fluoxetine and bone loss. Depression is a major contributor to the deterioration of biomechanical performance, such as elastic energy absorption and 3-point bending strength. In addition, there were no significant differences in femur microarchitecture parameters between fluoxetine-only and control groups. Recovery of biomechanical strength has been observed after 16 weeks of fluoxetine treatment. Fluoxetine treatment led to significantly higher bone volume, trabecular bone volume, a higher number of osteoblasts per bone area, energy absorption and bending strength in rats without exposure to stress.

Previous human studies could not remove confounding factors, such as the presence of smoking, substance abuse, fall risk, body mass index, ethnicity, diet, and the use of other medications. The foregoing confounding factors cause a spurious relationship between SSRI use and bone loss in previous studies. For example, SSRIs inhibit cardiovascular sodium and calcium channels, leading to dysrhythmia and increase in fall and fracture risk without lowering BMD [48]. Clinicians have to be cautious not to associate SSRI use with bone loss in order to minimize nonadherence to treatment and unnecessary anxiety.

## Conclusion

In conclusion, fluoxetine did not induce the deterioration of biomechanical performance under the CMS model of depression. Without exposure to stress, fluoxetine increased average midshaft thickness, osteoblast per bone area and bending strength in rats without exposure to CMS. Under the CMS, both CMS + fluoxetine and CMS + placebo showed the deterioration of bone structure, such as increase in trabecular separation, decrease in trabecular number and higher resorption parameter. There were no statistical significance differences in bone microarchitecture in rats exposed to fluoxetine and control condition without stress. Previous reports describing the association between fluoxetine and low BMD in humans suffering from depression were susceptible to confounding by other factors, such as age, ethnicity, variation in diet and nutrition, smoking, substance abuse and concurrent use of other medications.

## Supplementary information

Supplementary figure 1.

Supplementary Table 1

Supplementary Table 2

Supplementary Table 3

Supplementary Table 4

Supplementary Table 5
