# Supplementary figures and images for: Fluoxetine improves bone microarchitecture and mechanical properties in rodents undergoing chronic mild stress – an animal model of depression
Source: Transl Psychiatry. 2022 Aug 20;12:339. doi: 10.1038/s41398-022-02083-w (PMC9392792; doi:10.1038/s41398-022-02083-w)

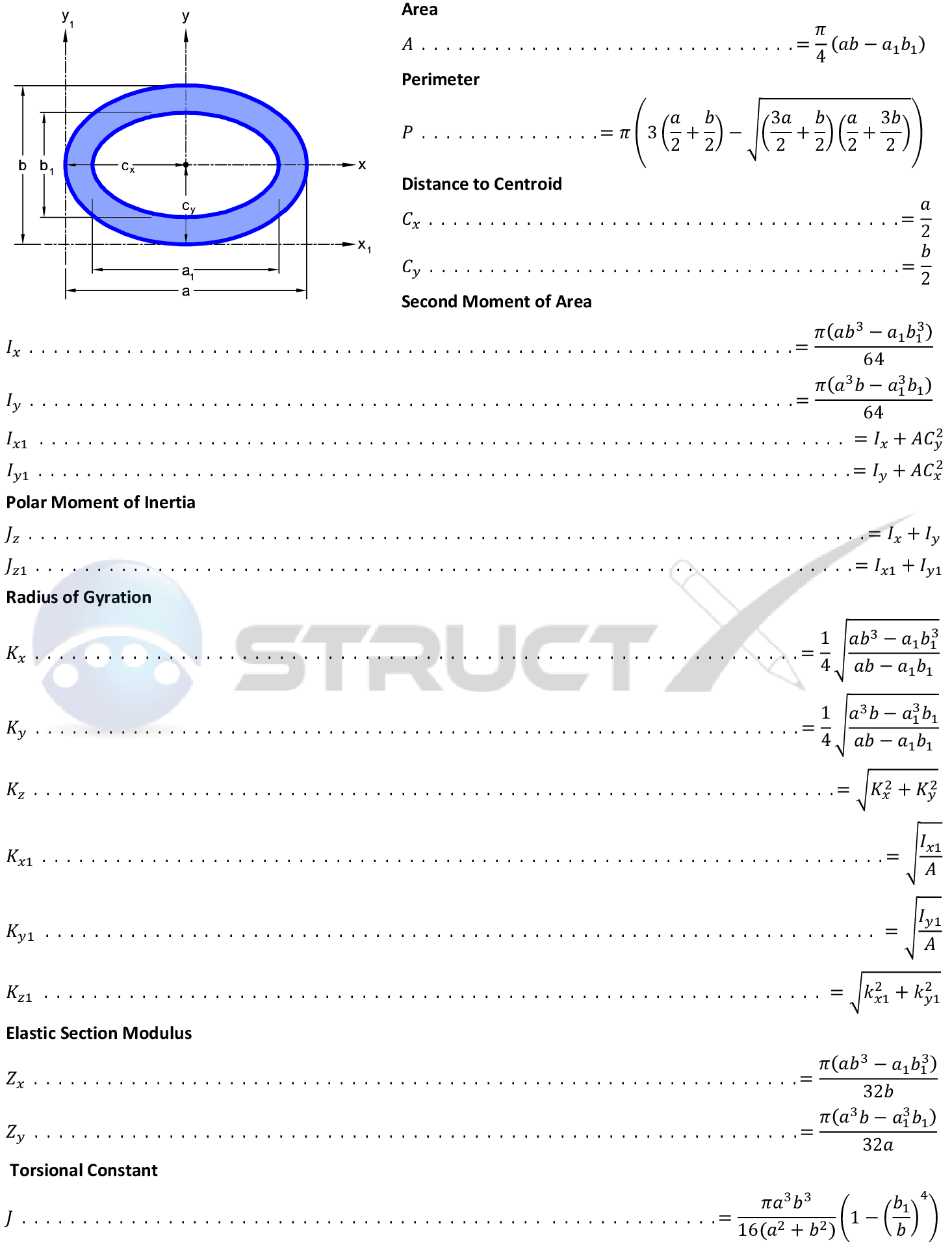


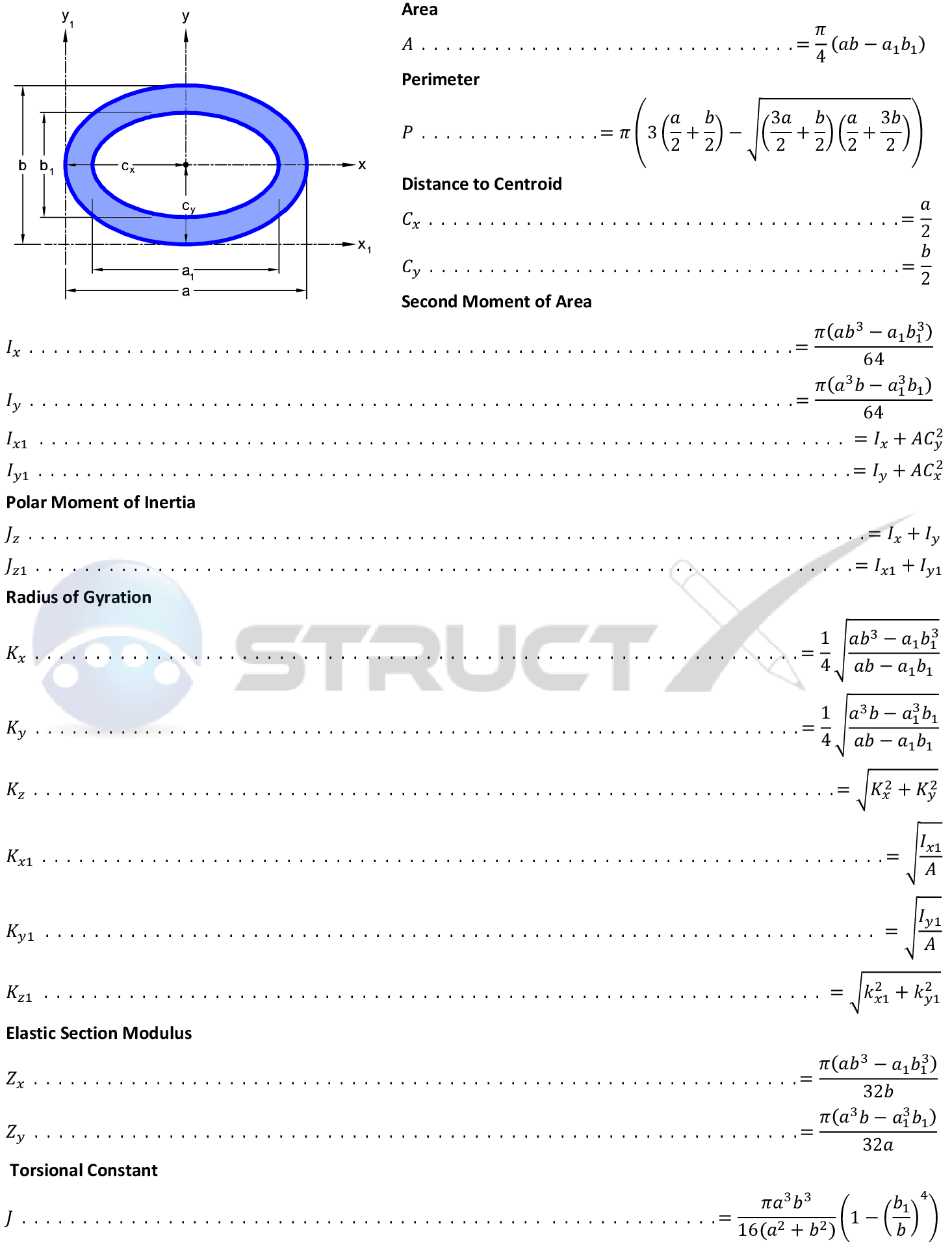

Supplement: Supplementary file 1 — Supplementary figure 1. [file 41398_2022_2083_MOESM1_ESM.docx]
