# Supplementary material for: Fluoxetine improves bone microarchitecture and mechanical properties in rodents undergoing chronic mild stress – an animal model of depression
Source: Transl Psychiatry. 2022 Aug 20;12:339. doi: 10.1038/s41398-022-02083-w (PMC9392792; doi:10.1038/s41398-022-02083-w)
Supplement: Supplementary file 2 — Supplementary Table 1 [file 41398_2022_2083_MOESM2_ESM.docx]

**Supplementary Table 1 Pearson correlation between volume (vol) of sucrose intake and** **bone histomorphometry parameters**

|  | Control |  | Fluoxetine-only |  | CMS+ placebo |  | CMS +fluoxetine |  |
| --- | --- | --- | --- | --- | --- | --- | --- | --- |
| Correlation | Pearson Correlation | p-value | Pearson Correlation | p-value | Pearson Correlation | p-value | Pearson Correlation | p-value |
| Vol and BV/TV% | -0.493 | 0.398 | -0.938 | 0.019* | -0.013 | 0.974 | 0.180 | 0.619 |
| Vol and BS/BV% | 0.618 | 0.267 | 0.396 | 0.509 | 0.121 | 0.757 | -0.223 | 0.537 |
| Vol and BS/TV% | 0.421 | 0.480 | -0.335 | 0.582 | -0.226 | 0.529 | -0.063 | 0.863 |
| Vol and Tb.Th | -0.791 | 0.111 | -0.335 | 0.582 | -0.226 | 0.529 | -0.062 | 0.863 |
| Vol and Tb.Sp | 0.761 | 0.135 | -0.129 | 0.836 | -0.012 | 0.996 | 0.398 | 0.254 |
| Vol and Tb.N | 0.690 | 0.198 | -0.078 | 0.900 | 0.225 | 0.560 | 0.138 | 0.704 |

*p<0.05, BV/TV, % = Trabecular bone volume; BS/BV % = bone surface/bone volume; BS/TV % = bone surface/tissue volume; Tb.Th = The trabecular thickness; Tb.N =trabecular number and Tb.Sp = trabecular separation
