# Supplementary material for: Fluoxetine improves bone microarchitecture and mechanical properties in rodents undergoing chronic mild stress – an animal model of depression
Source: Transl Psychiatry. 2022 Aug 20;12:339. doi: 10.1038/s41398-022-02083-w (PMC9392792; doi:10.1038/s41398-022-02083-w)
Supplement: Supplementary file 3 — Supplementary Table 2 [file 41398_2022_2083_MOESM3_ESM.docx]

**Supplementary Table 2 Pearson correlation between immobility time (IT) during the forced swim test and histomorphometry parameters**

|  | Control |  | Fluoxetine-only |  | CMS + placebo |  | CMS + fluoxetine |  |
| --- | --- | --- | --- | --- | --- | --- | --- | --- |
| Correlation | Pearson Correlation | p-value | Pearson Correlation | p-value | Pearson Correlation | p-value | Pearson Correlation | p-value |
| IT and BVTV | 0.164 | 0.756 | -0.514 | 0.375 | 0.391 | 0.338 | 0.697 | 0.082 |
| IT and BSBV | 0.090 | 0.865 | 0.092 | 0.883 | 0.258 | 0.537 | 0.395 | 0.380 |
| IT and BSTV | 0.735 | 0.157 | -0.465 | 0.430 | 0.399 | 0.328 | 0.680 | 0.093 |
| IT and Tb.Th | -0.194 | 0.712 | -0.136 | 0.827 | -0.228 | 0.588 | -0.477 | 0.279 |
| IT and Tb.Sp | 0.017 | 0.974 | 0.270 | 0.661 | -0.709 | 0.049* | -0.647 | 0.116 |
| IT and Tb.N | 0.585 | 0.223 | -0.044 | 0.944 | 0.569 | 0.141 | 0.798 | 0.032* |

*p<0.05, BV/TV, % = Trabecular bone volume; BS/BV % = bone surface/bone volume; BS/TV % = bone surface/tissue volume; Tb.Th = The trabecular thickness; Tb.N =trabecular number and Tb.Sp = trabecular separation
