# Supplementary material for: Fluoxetine improves bone microarchitecture and mechanical properties in rodents undergoing chronic mild stress – an animal model of depression
Source: Transl Psychiatry. 2022 Aug 20;12:339. doi: 10.1038/s41398-022-02083-w (PMC9392792; doi:10.1038/s41398-022-02083-w)
Supplement: Supplementary file 4 — Supplementary Table 3 [file 41398_2022_2083_MOESM4_ESM.docx]

**Supplementary Table 3 Pearson correlation between the volume (Vol) of sucrose Intake and biomechanics parameters**

|  | **Control** |  | **Fluoxetine-only** |  | **CMS + placebo** |  | **CMS+ fluoxetine** |  |
| --- | --- | --- | --- | --- | --- | --- | --- | --- |
| Correlation | Pearson Correlation | p-value | Pearson Correlation | p-value | Pearson Correlation | p-value | Pearson Correlation | p-value |
| Vol and Elastic stiffness | -0.606 | 0.279 | 0.793 | 0.109 | -0.086 | 0.825 | -0.412 | 0.270 |
| Vol and Elastic Absorption Energy | 0.141 | 0.821 | 0.999 | 0.001* | -0.201 | 0.604 | 0.129 | 0.783 |

*p<0.05
