# Supplementary material for: Fluoxetine improves bone microarchitecture and mechanical properties in rodents undergoing chronic mild stress – an animal model of depression
Source: Transl Psychiatry. 2022 Aug 20;12:339. doi: 10.1038/s41398-022-02083-w (PMC9392792; doi:10.1038/s41398-022-02083-w)
Supplement: Supplementary file 5 — Supplementary Table 4 [file 41398_2022_2083_MOESM5_ESM.docx]

**Supplementary Table 4 Pearson Correlation between immobility time (IT) during the forced swim test and biomechanics parameters**

|  | **Control** |  | **Fluoxetine-only** |  | **CMS+placebo** |  | **CMS+fluoxetine** |  |
| --- | --- | --- | --- | --- | --- | --- | --- | --- |
| Correlation | Pearson Correlation | p-value | Pearson Correlation | p-value | Pearson Correlation | p-value | Pearson Correlation | p-value |
| IT and Elastic stiffness | -0.177 | 0.776 | 0.146 | 0.815 | 0.297 | 0.474 | 0.178 | 0.703 |
| IT and Elastic Absorption Energy | -0.493 | 0.399 | 0.502 | 0.498 | 0.194 | 0.645 | -0.124 | 0.791 |
