# Supplementary material for: Fluoxetine improves bone microarchitecture and mechanical properties in rodents undergoing chronic mild stress – an animal model of depression
Source: Transl Psychiatry. 2022 Aug 20;12:339. doi: 10.1038/s41398-022-02083-w (PMC9392792; doi:10.1038/s41398-022-02083-w)
Supplement: Supplementary file 6 — Supplementary Table 5 [file 41398_2022_2083_MOESM6_ESM.docx]

**Supplementary Table 5 Comparison of pro-inflammatory cytokine levels (4-week vs baseline; 16-week vs baseline) in the CMS-fluoxetine, CMS-placebo and control groups**

| **IL-1β** | **Baseline** | **4-week** | **4-week vs baseline**  **p-value** | **16-week** | **16-week vs baseline**  **p-value** |
| --- | --- | --- | --- | --- | --- |
| CMS Fluoxetine | 1.31 ±2.18 | 8.85 ±12.03 | 0.3698 | 4.95 ±8.10 | 0.9720 |
| CMS Placebo | 0.00 ±0.00 | 0.61 ±1.35 | 1 | 4.10 ±8.11 | 0.9444 |
| Control | 3.39 ±1.41 | 0.98 ±1.41 | 0.5683 | 10.21 ± 16.21 | 0.8715 |

| **IL-6** | **Baseline** | **4-week** | **4-week vs baseline**  **p-value** | **16-week** | **16-week vs baseline**  **p-value** |
| --- | --- | --- | --- | --- | --- |
| CMS Fluoxetine | 308.54 ±708.58 | 145.58 ±310.17 | 1 | 333.09 ±392.88 | 1 |
| CMS Placebo | 739.36 ±1461.84 | 67.57 ±145.07 | 0.7128 | 814.45 ±1227.72 | 1 |
| Control | 294.23 ±657.91 | 113.05 ±163.95 | 1 | 260.70 ±222.03 | 0.9833 |

| **IL-17** | **Baseline** | **4-week** | **4-week vs baseline**  **p-value** | **16-week** | **16-week vs baseline**  **p-value** |
| --- | --- | --- | --- | --- | --- |
| CMS Fluoxetine | 7.48 ±18.63 | 1.61 ±3.18 | 1 | 10.07 ±9.48 | 1 |
| CMS Placebo | 23.64 ±48.89 | 3.12 ± 5.89 | 1 | 107.18 ±225.41 | 0.6162 |
| Control | 0.01 ±0.02 | 34.43 ± 76.98 | 1 | 118.02 ±181.13 | 0.6178 |

|  | |
| --- | --- |
|  |  |
|  |  |
|  |  |
|  |  |
